# IL‐17A is a pertinent therapeutic target for moderate‐to‐severe hidradenitis suppurativa: Combined results from a pre‐clinical and phase II proof‐of‐concept study

**DOI:** 10.1111/exd.14619

**Published:** 2022-08-19

**Authors:** Alexa B. Kimball, Christian Loesche, Errol P. Prens, Falk G. Bechara, Jamie Weisman, Izabela Rozenberg, Philip Jarvis, Thomas Peters, Lukas Roth, Grazyna Wieczorek, Frank Kolbinger, Gregor B. E. Jemec

**Affiliations:** ^1^ Harvard Medical School and Clinical Laboratory for Epidemiology and Applied Research in Skin (CLEARS), Department of Dermatology Beth Israel Deaconess Medical Center Boston Massachusetts USA; ^2^ Novartis Institutes for BioMedical Research Novartis Pharma AG Basel Switzerland; ^3^ Department of Dermatology Erasmus University Medical Centre Rotterdam Netherlands; ^4^ Department of Dermatology, Venereology and Allergology Ruhr‐University Bochum Bochum Germany; ^5^ Advanced Medical Research Atlanta Georgia USA; ^6^ Department of Dermatology, Zeeland University Hospital, Roskilde, Health Sciences Faculty University of Copenhagen Roskilde Denmark

**Keywords:** clinical trial, hidradenitis suppurativa, IL‐17A, pathophysiology, T cells

## Abstract

Hidradenitis Suppurativa (HS) is a chronic, recurrent, inflammatory, follicular skin disease whose pathology is complex and not fully understood. The objective of this study was to elucidate the role of IL‐17A in moderate‐to‐severe HS. Transcriptomic and histological analyses were conducted on ex vivo HS (*n* = 19; lesional and non‐lesional) and healthy control (*n* = 8) skin biopsies. Further, a Phase II exploratory, randomized, double‐blind, placebo‐controlled study was carried out in moderate‐to‐severe HS patients. Patients were treated with either CJM112 300 mg (*n* = 33), a fully human anti‐IL‐17A IgG1/κ monoclonal antibody, or placebo (*n* = 33). The main outcome of the translational analyses was to identify IL‐17A‐producing cells and indications of IL‐17A activity in HS lesional skin. The primary objective of the clinical study was to determine the efficacy of CJM112 in moderate‐to‐severe HS patients by HS‐Physician Global Assessment (HS‐PGA) responder rate at Week 16. Transcriptomic and histopathologic analyses revealed the presence of heterogeneous cell types in HS lesional skin; IL‐17A gene signatures were increased in HS lesional vs non‐lesional or healthy skin. High expression of IL‐17A was localized to T cells, neutrophils, and mast cells, confirming the transcriptional data. Clinically, the proportion of Week 16 HS‐PGA responders was significantly higher (*p* = 0.03) in the CJM112 group vs placebo (32.3% vs 12.5%). This study elucidated the role of the IL‐17A pathway in HS pathogenesis and clinically validated the IL‐17A pathway in moderate‐to‐severe HS patients in a proof‐of‐concept study using the anti‐IL‐17A‐specific antibody CJM112.

## 1 INTRODUCTION

Hidradenitis suppurativa (HS) is a painful, chronic, recurrent, inflammatory, follicular skin disease,^1^, ^2^ characterized by deep dermal inflammatory nodules and abscesses.^1^ Inflammatory lesions may progress to tunnel formation and sometimes fistulization, both of which are associated with pain, hypertrophic scarring, and possible impact on functional use of the limbs.^3^, ^4^ Epidemiologic studies report a varying global prevalence between 0.1 and 4%^5^, ^6^, ^7^; these inconsistencies may be due to significant delays of 7–10 years between disease onset and diagnosis,^8^, ^9^ under‐reporting by patients due to embarrassment and shame associated with HS^10^ or differences in prevalence due to ethnicity.^11^ HS is a disease with a high unmet medical need that has a devastating effect on patients' quality of life (QoL).

HS treatment is individualized and includes lifestyle alterations (e.g. smoking cessation), topical and systemic antibiotic therapy, immunomodulatory agents as well as localized and extensive surgical interventions.^12^ Treatment decisions are based on criteria including, disease severity, anatomical location, and comorbidity profile.

The pathophysiology of HS is not fully understood but it appears to involve immune system dysregulation, altered microbiome and hormonal, genetic and environmental influences. Transcriptomic and lipidomic analyses of HS biopsies revealed a dysregulation of several inflammatory pathways,^13^, ^14^ including dysregulation of T helper‐type 17 cytokines. Recent translational studies have indicated more specifically that the interleukin (IL)‐17 pathway is a potential target for the effective treatment of moderate‐to‐severe HS^15^: Th17 cells are enriched in HS lesional skin,^16^ IL‐17A expression is elevated in HS compared with healthy skin,^17^, ^18^ and serum IL‐17A levels are also increased and correlate with HS disease severity.^19^

Based on these data, targeting the IL‐17 pathway may be beneficial in the treatment of moderate‐to‐severe HS. Several case reports and small open‐label studies have demonstrated positive results with secukinumab and ixekizumab (both monoclonal antibodies which neutralize IL‐17A), bimekizumab (monoclonal antibody neutralizing both IL‐17A and IL‐17F) and brodalumab (IL‐17A receptor‐antagonist).^20^, ^21^, ^22^, ^23^, ^24^, ^25^, ^26^, ^27^, ^28^

In this study, we combine ex vivo and in vivo clinical methodologies to elucidate the role of IL‐17A in moderate‐to‐severe HS. Using combined transcriptomic and histological analysis of ex vivo lesional and non‐lesional HS tissue biopsies, we confirmed that HS skin pathology is in part driven by Th17 cells, neutrophils, and macrophages. IL‐17A‐positive cells are found in both the lesional and non‐lesional skin of moderate‐to‐severe HS patients, with a prominent IL‐17A gene signature identified in HS lesion compared with non‐lesional skin. Finally, using CJM112, a novel human anti‐IL‐17A high‐affinity IgG1/κ monoclonal antibody, we demonstrate the clinical utility of anti‐IL‐17A therapy in a HS proof‐of‐concept Phase II randomized clinical trial.

## 2 METHODS

### 2.1 Translational study

#### 2.1.1 Translational study objective

The objective of the translational research was to identify IL‐17A‐producing cell types, find evidence of IL‐17A activity and correlate HS to a disease in which IL‐17A inhibition is an effective treatment (psoriasis). Information on sample collection, transcriptomics, and immunohistochemical analyses is extensively described in the Appendix S1.

### 2.2 Clinical study

#### 2.2.1 Clinical study design

This clinical study was a Phase II, exploratory, randomized, double‐blind, placebo‐controlled, multicentre study in patients with moderate‐to‐severe HS (clinicaltrials.gov identifier: NCT02421172). The investigational medicinal product (IMP) utilized in this proof‐of‐concept study was CJM112, a novel fully human anti‐IL‐17A IgG1/κ monoclonal antibody which binds with similar affinity to both IL‐17A and IL‐17AF. CJM112 previously demonstrated clinical utility in a First‐in‐Human (FIH) study in moderate‐to‐severe psoriasis patients.^29^

The study was carried out in two treatment periods, Treatment Period 1 (16 weeks; TP1) and an exploratory Extension Period 2 (16 weeks; EP2) followed by a 12‐week treatment‐free follow‐up period. In the FIH study by Kaul et al., subcutaneous (s.c.) CJM112 150 mg was clinically effective in moderate‐to‐severe psoriasis patients, with no safety signals identified in doses up to 450 mg.^29^ Thus, a dose of subcutaneous CJM112 300 mg was selected for this study to maximize dosing in a difficult‐to‐treat disease. Patients received a total of 10 doses; the first five doses were administered weekly, followed by injections every other week (q2w) until Week 16.

Previous clinical trial data were used to estimate the effect of placebo on clinical outcomes; based on Kimball et al., it was assumed that HS severity over time without treatment does not change significantly (approximately 5% change in HS Physician's Global Assessment scores [HS‐PGA]).^30^ Therefore, patients were not re‐randomized after the end of TP1 (Week 16). EP2 was designed to provide all patients included with the opportunity to receive the IMP. Patients receiving CJM112 300 mg in TP1 were switched to receive placebo and patients receiving placebo were switched to receive either CJM112 300 mg or CJM112 50 mg at Week 16. Here, we focus on Week 16 data only, which includes the primary and key secondary endpoints.

#### 2.2.2 Clinical study objectives

The primary objective of this study was to determine the efficacy of CJM112 300 mg in moderate‐to‐severe HS patients by HS‐PGA responder rate (≥2‐point reduction in HS‐PGA score) at Week 16. Additional exploratory efficacy endpoints included measuring absolute and percent change from the Baseline in: HS inflammatory lesion count (abscesses, nodules, draining tunnels) and the HS Clinical Response score (HiSCR). High‐sensitivity C‐reactive protein (hsCRP) was measured in blood samples from HS patients as a surrogate marker of systemic inflammation. The Dermatology Life Quality Index (DLQI) was measured to assess the effect of CJM112 on patient‐reported outcomes. Further objectives of this study included safety and tolerability, pharmacodynamics and pharmacokinetics of CJM112.

Additional methodologies are described further in Appendix S1.

### 2.3 Ethics

Both translational and clinical studies were conducted in accordance with the International Conference on Harmonization Guidelines for Good Clinical Practice and the Declaration of Helsinki. All patients provided written informed consent before participation in either the translational or clinical study. The non‐interventional biomarker study and the clinical study protocols were approved by the Institutional Review Board or Independent Ethics Committee of each participating centre.

## 3 RESULTS

### 3.1 Translational study

#### 3.1.1 Transcriptomic analysis demonstrates IL‐17‐pathway activity in HS lesional skin

**FIGURE 1:**
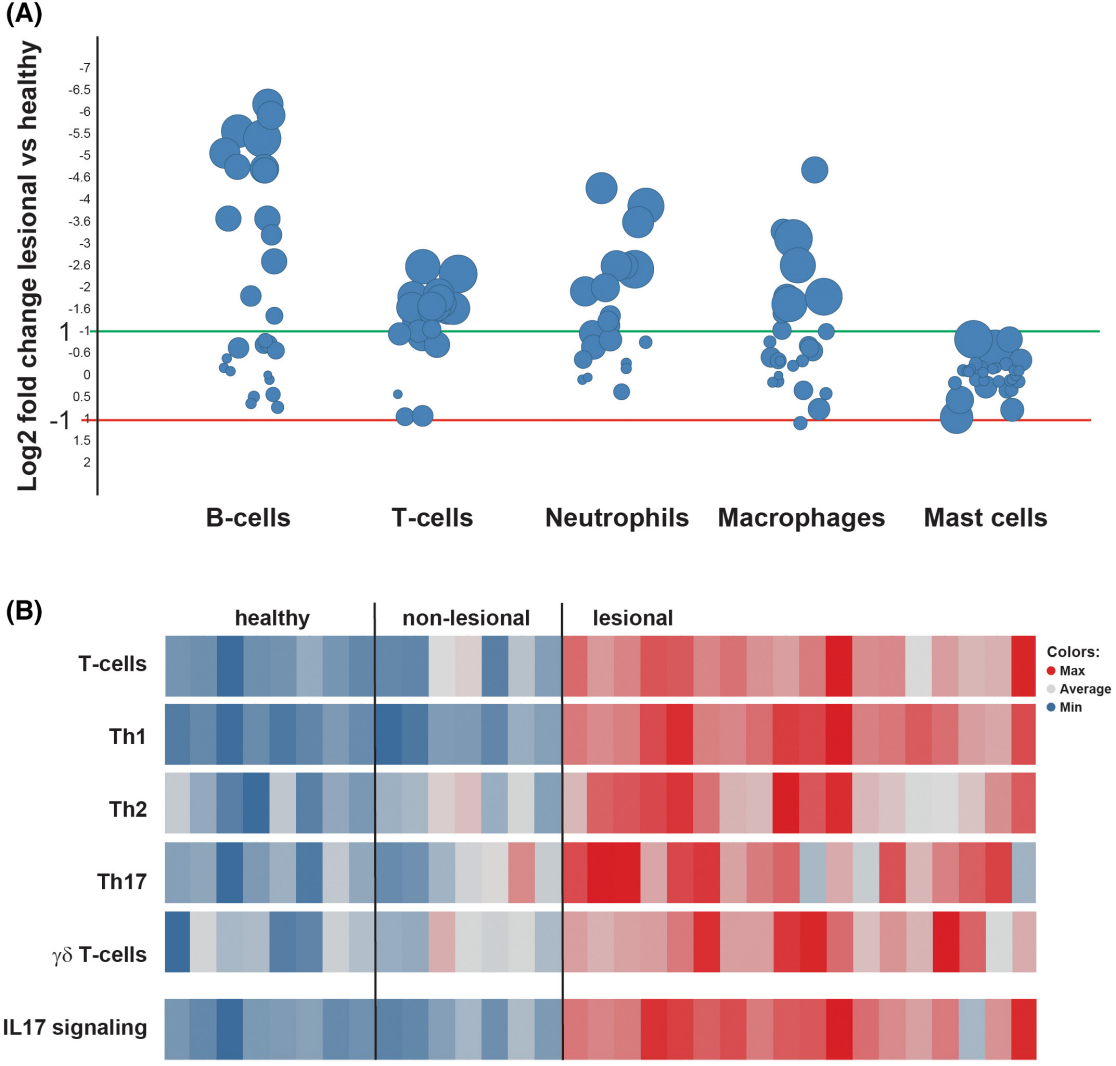
Transcriptional Analysis of Lesional HS Biopsies. (A) Dot plot showing the log_2_ fold change between lesional HS vs healthy control for individual donors (one dot is one gene) of a cell type signature. The size is by log adjusted *p*‐value (the bigger the size, the lower the *p*‐value). (B) Median expression levels of defined T‐cell types and IL‐17A signalling pathways are visualized in the heat map. Each block depicts the color‐graded level of the expression in this particular RNA sample with blue showing the minimal and red the maximal level for the respective gene set. HS, hidradenitis suppurativa; IL, interleukin; RNA, ribonucleic acid; Th, T helper

To investigate which pathways and cell types were active in lesional HS, we performed an Affymetrix‐based transcriptomics analysis. Differential gene expression analysis revealed a large number of dysregulated genes. To deconvolute the full tissue biopsy for prevalent cell types, we used a highly distinctive set of genes previously published.^31^, ^32^ Cell types identified in HS lesions included neutrophils, macrophages, B cells, and various T‐cell subsets such as Th1 and Th17 cells (Figure 1A,B). T‐cell subsets, Th17, and γδT cells are reported to produce IL‐17A in other skin diseases (e.g. psoriasis), which in turn can elicit a signalling cascade in the epidermis and dermis.^18^, ^33^

IL‐17A gene signatures based on IL‐17A stimulation in various cell types (keratinocytes, fibroblasts, whole skin) showed an increase in median expression levels in HS lesional skin compared with healthy or HS non‐lesional skin, suggesting IL‐17A pathway engagement in HS (Figure 1B).

**FIGURE 2:**
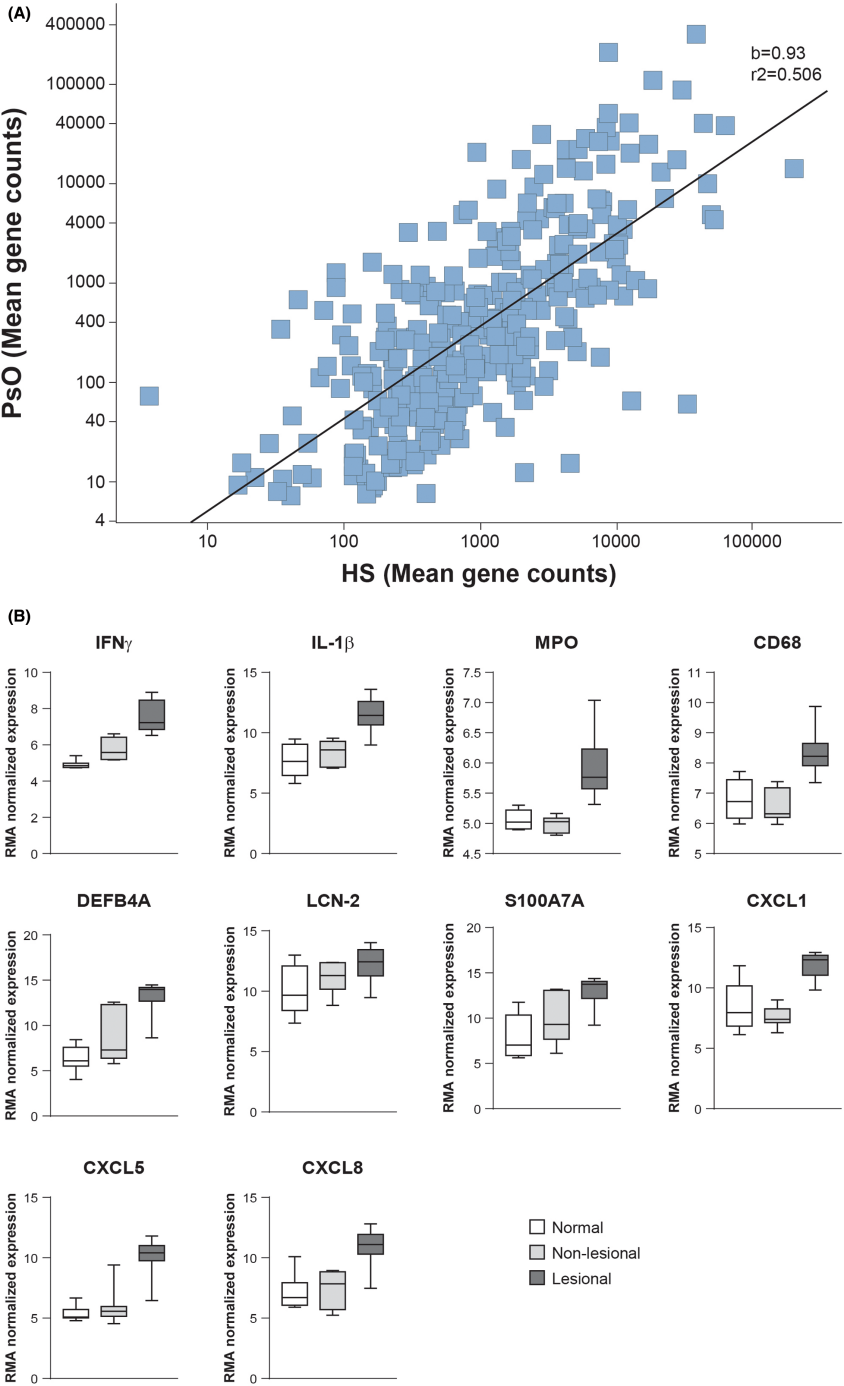
Analysis and Comparison of HS and Psoriasis Lesional Biopsies. (A) Nanostring data analysis showing a correlation between PsO and HS expression levels of selected genes. The black line represents the linear regression, *r* ^2^ (correlation coefficient) is 0.506, and b (slope of the line) is 0.93. (B) Boxplots demonstrating Robust Multichip Average (RMA) normalized data of key genes analysed using GeneSpring (11.5.1) Boxplots represent min, max, median, and upper and lower quartiles. CD, cluster of differentiation; CXCL, chemokine C‐X‐C motif ligand 1; DEF4BA, beta‐defensin 4A precursor; HS, hidradenitis suppurativa; IFN, interferon; IL, interleukin; LCN‐2, lipocalin 2; MPO, myeloperoxidase; PsO, psoriasis.; RMA, Robust Multichip Average; S100A7A, S100calcium‐binding protein A7A

An independent, more focused analysis using a custom panel of inflammation and psoriasis‐related genes showed a good correlation of expression levels between lesional psoriasis samples and lesional HS samples (Figure 2A).

Genes of interest identified in NanoString analysis were further validated using the Affymetrix dataset (Figure 2B). Key candidate genes involved in psoriasis pathogenesis and linked to the IL‐17A pathway were markedly overexpressed in HS lesional skin compared to non‐lesional and healthy skin, including *DEFB4A*, *LCN2*, *S100A7A*, *CXCL8*, and *CXCL5* (**all** Figure 2B).

In addition, unbiased pathway analysis of differentially expressed genes between lesional HS and healthy volunteer samples identified the IL‐17A pathway as one of the top canonical pathways using Ingenuity Pathway Analysis tool (IPA analysis, Figure S1A). Similarly, various members of the IL‐17 ligand/receptor family were identified as upstream regulators in this analysis (Figure S1B).

#### 3.1.2 IL‐17A is highly expressed in HS lesional skin and is localized to T cells, neutrophils and mast cells

**FIGURE 3:**
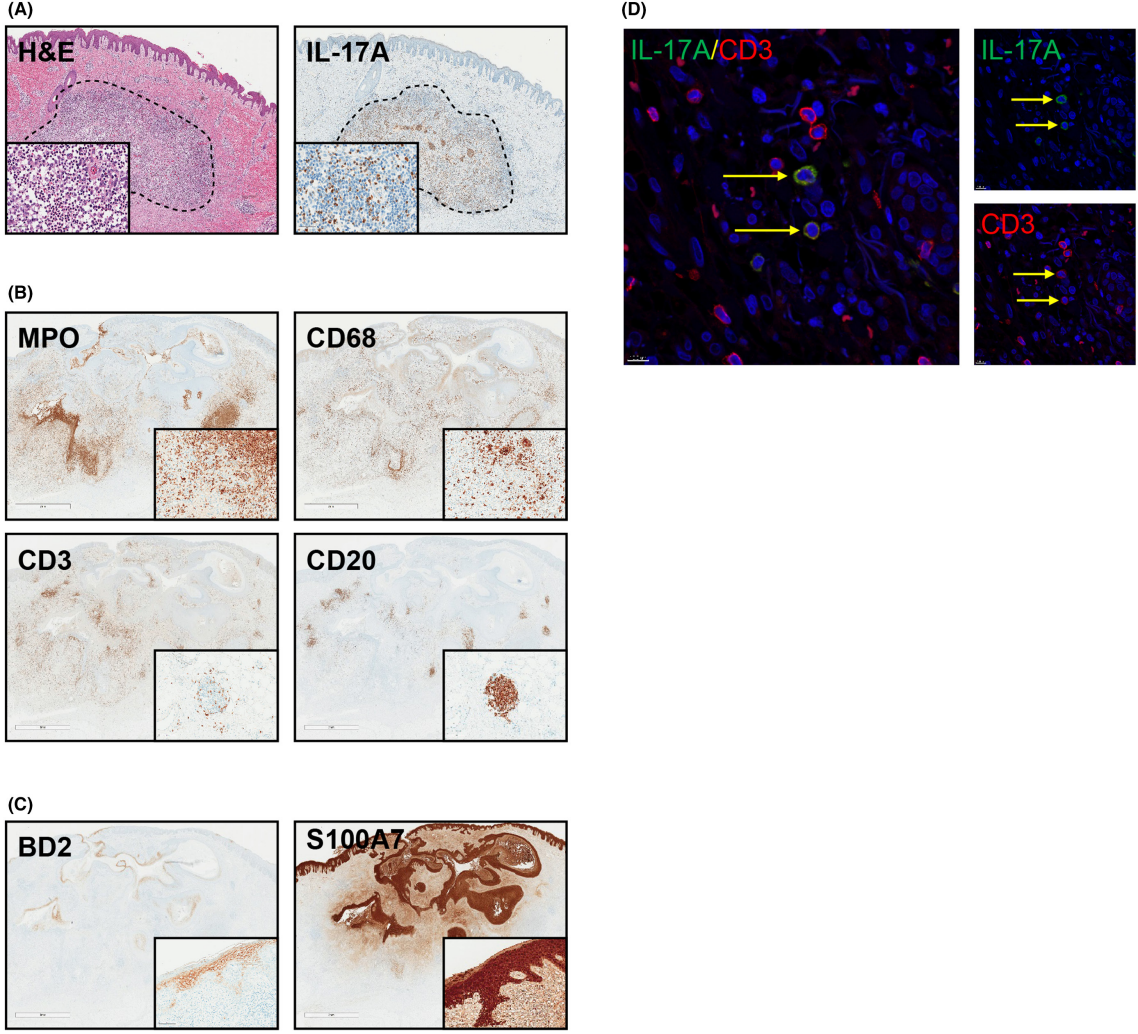
Immunohistochemical Analysis of HS Skin Biopsies. (A) Representative image of HS lesional biopsy showing dermal inflammation with abscess formation and IL‐17A‐positive cells. (B) Dermal lesions containing numerous neutrophils (MPO) and macrophages (CD68) as well as clusters of T (CD3) and B cells (CD20). (C) Strong epidermal expression of BD‐2 and S100A7/psoriasin above the inflammatory dermal lesions. (D) Double immunofluorescence illustrating the presence of CD3+/IL‐17+ positive cells in the dermis. BD‐2, beta‐defensin 2; H&E, haematoxylin, and eosin; IL, interleukin; MPO, myeloperoxidase; S100A7, S100 calcium‐binding protein A7

Histopathology revealed mixed inflammatory infiltrates consisting of mainly macrophages, neutrophils, and T and B cells in the lesional biopsies. A high expression of IL‐17A was observed in the lesional biopsies localized to T cells, neutrophils, and mast cells, thus confirming our transcriptional data (Figure 3A,B). The inflammatory cells were located in the dermis while β defensin 2 (BD‐2) and S100A7/psoriasin were both found in areas of psoriasiform epidermal hyperplasia, above the dermal infiltrates (Figure 3C). Double immunofluorescence staining revealed the presence of IL‐17A^+^CD3^+^ cells in the lesional infiltrate in the dermis (Figure 3D).

### 3.2 Clinical study

#### 3.2.1 Overview of Phase II clinical trial patient population

In total, 66 patients were randomized (33 patients each in CJM112 and placebo groups), of which 60 (90.9%) completed Week 16 (primary endpoint; TP1). Of these, 50 patients (83.3%) completed EP2 and 49 (81.7%) completed the 12‐week follow‐up period. The most common reason for study discontinuation at Week 16 was a loss of follow‐up (4/6 patients). Additional patient discontinuations were either due to an adverse event (AE; cystitis, 1/6) or patient decision (1/6). A CONSORT flow graph demonstrating patient disposition is illustrated in Figure S2.

Baseline demographics and disease characteristics are presented in Table S1. Overall, baseline demographics were comparable between treatment groups and were typical of a chronic HS population. Of note, a higher baseline inflammatory lesion count and more severe Hurley scores were observed in the CJM112 treatment group compared with placebo patients. Mean hsCRP levels were much higher at baseline (20.1 ± 20.05 mg/L) than the normal range (0–5 mg/L).

#### 3.2.2 IL‐17‐blockade with CJM112 demonstrates clinical efficacy in patients with moderate‐to‐severe HS

**FIGURE 4:**
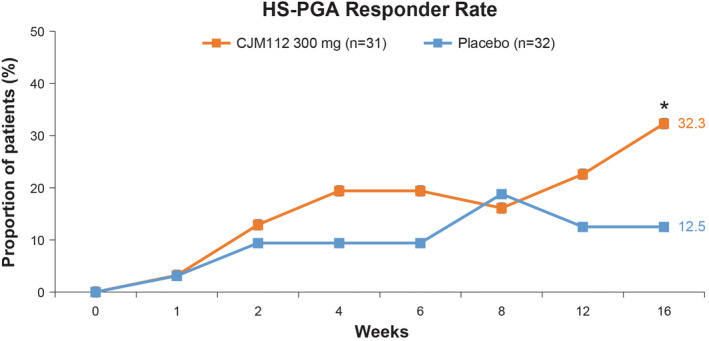
HS‐PGA Responder Rate (primary endpoint data). Line graph demonstrating HS‐PGA responder rate from Baseline to Week 16 in CJM112 300 mg and placebo‐treated HS patients. *p < 0.05 statistically different from placebo. HS‐PGA, hidradenitis suppurativa Physician's Global Assessment

At Week 16, the proportion of HS‐PGA responders was significantly higher in the CJM112 300 mg treatment group compared to placebo (32.3% [10/31] vs 12.5% [4/32]; *p* = 0.03) (Figure 4). This study met one of its primary pre‐defined dual efficacy criteria; CJM112 300 mg showed a superior treatment effect with a higher HS‐PGA responder rate than placebo, with a posterior probability of superiority being 97% (difference between groups 0.194 [95% credibility interval: −0.004; 0.392]). Although there was a statistically significant higher proportion of responders in the CJM112 300 mg treatment group compared with the placebo, the target efficacy difference in favour of CJM112 300 g of at least 30%, with at least 60% posterior probability was not met (14.6%). The HS‐PGA responder rate was higher in the CJM112 300 mg group compared with the placebo at Weeks 2, 4, and 12, although no statistically significant differences were found between the CJM112 300 mg and placebo groups at these time points (Figure 4).

A noteworthy and unexpected decrease in the mean Baseline HS‐PGA score was observed at the start of EP2, following 16 weeks of placebo treatment. Since this placebo response was proportionately higher than what was assumed at protocol development (12.5% vs approximately 5%, respectively), and given that patients were not re‐randomized at Week 16 before entering EP2, interpretation of efficacy results beyond Week 16 were limited. Approximately 30% of patients had a very severe HS‐PGA score at baseline, while at Week 16 after placebo treatment, only approximately 20% of patients were severe. The median inflammatory lesion count at Week 16 was also lower in patients treated with placebo. The HS‐PGA responder rate of patients initially treated with CJM112 300 mg from Weeks 0–16 was variable, but was sustained overall from Weeks 16–32. However, possibly due to the lower severity at Week 16 after placebo treatment, no significant responder rates were observed in either placebo/CJM112 50 mg or placebo/CJM112 300 mg groups.

The mean total inflammatory lesion count was decreased in both CJM112 300 mg treated (21.0 vs 9.2 lesions [56.2% reduction]) and placebo‐treated patients (18.2 vs 12.7 lesions [30.2% reduction]) (Figure S3A). There were no significant reductions observed between CJM112 300 mg and placebo groups for the individual inflammatory lesion types at Week 16 (Figure S3B–D). Nonetheless, CJM112 300 mg had the greatest effect on inflammatory nodules, with 15.9 vs 6.4 inflammatory nodules reported at Baseline and Week 16 (change −9.5 inflammatory nodules [59.8% reduction]), respectively, compared with 13.0 vs 8.1 inflammatory nodules in placebo‐treated patients (change, −4.9 inflammatory nodules [37.7% reduction]) (Figure S3D).

**FIGURE 5:**
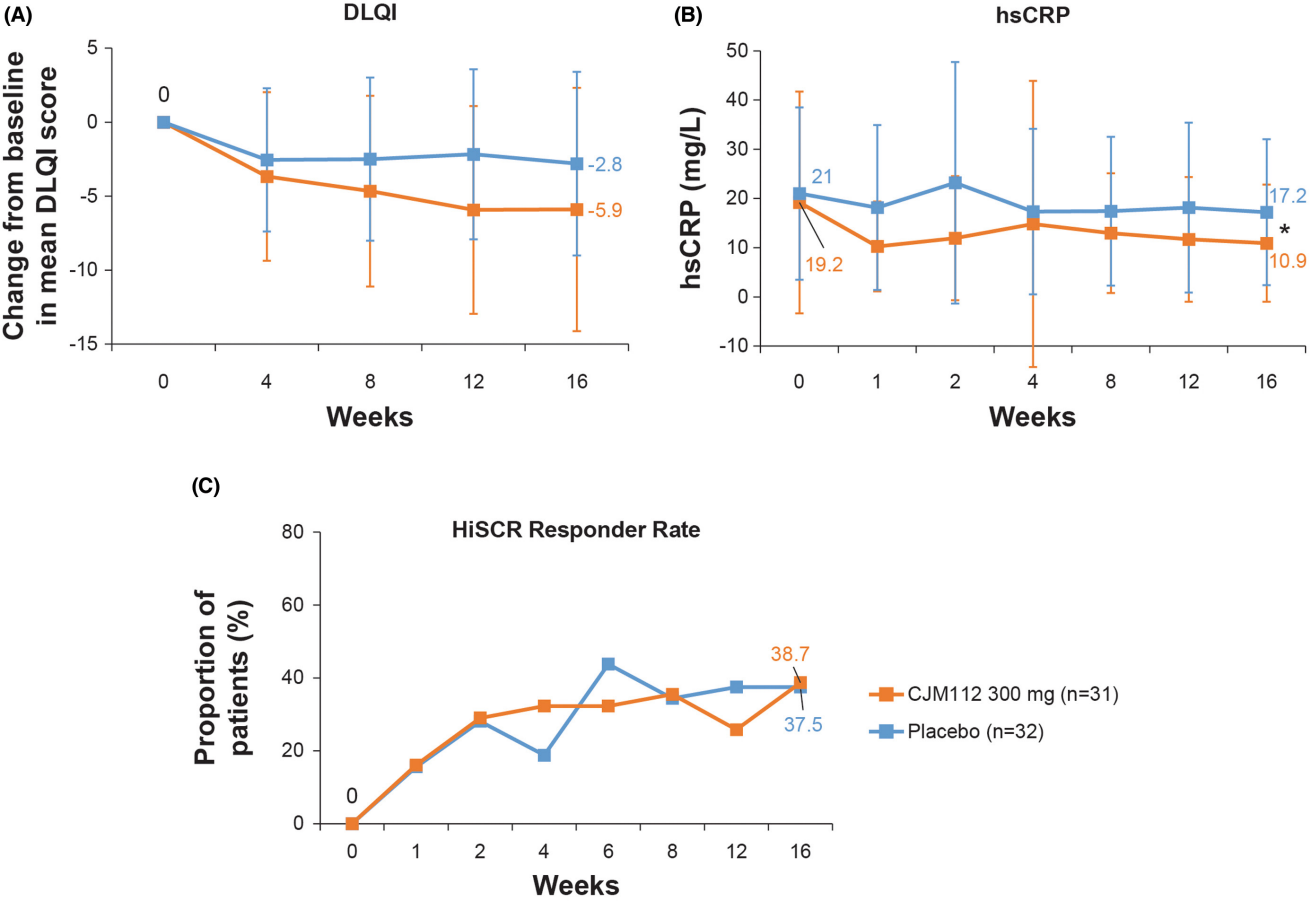
DLQI and hsCRP at Baseline Over Time to Week 16 in CJM112 300 mg and Placebo‐treated HS Patients. Line graphs demonstrating (A) mean change from Baseline in DLQI score, (B) mean hsCRP levels, and HiSCR responder rate from Baseline to Week 16 in CJM112 300 mg and placebo‐treated HS patients. Error bars represent standard deviation. *p < 0.05 statistically different from placebo. DLQI, Dermatology Life Quality Index; HiSCR, Hidradenitis Suppurativa Clinical Response; hsCRP, high sensitivity C‐reactive protein

The mean change in Dermatology Life Quality Index (DLQI) score from baseline to Week 16 showed higher reductions in patients who received CJM112 300 mg (−5.9 ± 8.23) compared with placebo (−2.8 ± 6.21) (Figure 5A). Mean hsCRP levels (previously observed as a soluble biomarker for the high inflammatory burden in HS^34^, ^35^, ^36^), were nearly 4‐fold higher at Baseline (19.2 mg/L and 21.0 mg/L for CJM112 and placebo, respectively), than normal range (≤5.0 mg/L), with a range of 0.4 mg/L to 82.4 mg/L. hsCRP was significantly decreased by CJM112 300 mg compared with placebo at Week 16 (10.9 vs 17.2 mg/L; *p* = 0.04) (Figure 5B). No change was demonstrated in Hidradenitis Suppurativa Clinical Response (HiSCR) responder rate between patients treated with CJM112 300 mg compared with placebo at Week 16, although this study was not powered for this analysis (Figure 5C).

#### 3.2.3 Supplementary Results

The pharmacokinetics and pharmacodynamics of CJM112 are extensively explored in *Kaul* et al.^29^ and briefly described in the Appendix S1.

Further, the frequency of AEs, most common AEs, and discontinuations due to AEs are described in the Appendix S1 and Table S2.

## 4 DISCUSSION

This investigation is comprised of both translational studies and a Phase II, proof‐of‐concept, clinical study which, taken together, suggest an important role for IL‐17A in HS pathogenesis, and indicate a role for IL‐17A‐targeting therapies in the treatment of moderate‐to‐severe HS (see graphical summary; Figure S4).

Due to the complex lesional topology in HS, a dataset combining transcriptomics and histological classification was generated, which identified Th17/IL‐17A pathway engagement and the presence of relevant cell types in lesional HS skin. Transcriptomic analysis demonstrates that specific Th17 cell signatures are overexpressed in lesional skin of HS patients compared with healthy skin. Furthermore, an IL‐17A‐induced gene signature was enriched in HS lesional skin, indicating IL‐17A‐induced downstream effects, which were not present in non‐lesional skin. Immunofluorescence data confirm this pattern, with increased T‐cell infiltration observed in HS lesional skin, and the presence of IL‐17A^+^CD3^+^ cells in lesional infiltrates, consistent with the premise that Th‐17 cells are a source of IL‐17A in HS disease, as previously demonstrated by Lima et al.^37^ These data support previous publications in which HS is reported as a Th1/Th17‐driven inflammatory disease, with a high frequency of Th17 cells in HS lesional skin, and increased production of IL‐17A not only in lesional HS skin, but also the peri‐lesional and uninvolved skin of HS patients.^17^, ^18^, ^38^, ^39^

Activation of the IL‐17A signalling pathway further induces the expression of pro‐inflammatory mediators including BD‐2, key S100 proteins, and chemokine ligands.^15^, ^40^ BD‐2 is a biomarker of IL‐17A‐driven pathology in psoriasis and S100A7 is associated with the severity of HS disease (Hurley staging), making these mediators useful surrogate markers of downstream IL‐17 activity.^41^ In this study we identified increased gene expression of *DEFB4A* (encoding BD‐2), *CXCL5*, *CXCL8*, *S100A7A* and *S100A9*, and increased BD‐2 and S100A7/ psoriasin protein expression in areas of psoriasiform epidermal hyperplasia mirroring dermal infiltrates, similar to previous findings.^37^, ^42^, ^43^

Consistent with our translational data which pointed to a pathogenic role of IL‐17A in HS, the current Phase II study demonstrated clinical efficacy of anti‐IL‐17A therapy with CJM112 in the treatment of moderate‐to‐severe HS. HS‐PGA was significantly reduced in patients treated with CJM112 300 mg compared with placebo at Week 16. Similar trends were seen in other endpoints in this pilot study, in particular, inflammatory lesion counts, although with numerical differences. CJM112 did not significantly decrease the HiSCR at Week 16 compared with placebo; however, this study was not powered to detect this difference. Other data support improvement in disease activity; the DLQI showed a severely impacted population prior to treatment, with a mean DLQI rate above 15 at Baseline. CJM112 300 mg improved the mean DLQI by approximately 6 points following 16 weeks of treatment. Mean hsCRP levels were elevated at Baseline, demonstrating the high inflammatory burden in HS patients, with CJM112 300 mg capable of significantly reducing hsCRP over 16 weeks of therapy.

This pilot study reported a higher placebo rate in the HS‐PGA response (12.5%) compared with similarly designed trials (*Kimball* et al. *2012* ^30^ [3.9%]; *Gottlieb* et al. *2016* ^44^ [5.6%]). Nonetheless, the current study reports a higher overall treatment effect over placebo (19.8% compared with 13.7% [*Kimball* et al. *2012* ^30^] and 13.8% [*Gottlieb* et al. *2016* ^44^]). High placebo rates have been frequently reported in other HS clinical studies, with heterogenous disease course, study design and patient experiences cited as key reasons for this occurrence.^45^ Better understanding of how draining tunnels are weighted and assessed in clinical trials will be important in the evolution of HS scoring systems.

At present, adalimumab, a human IgG1 monoclonal antibody that neutralizes tumor necrosis factor (TNF) α, is the only approved biologic treatment for chronic moderate‐to‐severe HS. The PIONEER I/II, Phase III double‐blind, placebo‐controlled, randomized controlled trials in chronic moderate‐to‐severe HS patients, demonstrated that approximately half of patients treated with adalimumab obtained a clinical response.^46^ As the pathophysiology of HS is still not well understood, mechanistic studies of HS biopsies are crucial to determine the role and interplay between cytokines to inform clinical decision‐making in HS management. Based on the data derived from mechanistic studies on HS pathogenesis, clinical studies are underway to assess the effects of inhibiting other potential targets in HS patients, including targets in the IL‐17 and JAK/STAT pathway, the complement cascade, and B cells.^47^

Since this randomized, controlled study was initiated, case reports and small open‐label studies with anti‐IL‐17A therapies have corroborated potential promise in the treatment of moderate‐to‐severe HS. In an open‐label trial of 9 HS patients previously failing antibiotic therapy, 67% achieved the primary outcome of HiSCR at Week 24, indicating at least a 50% improvement of total inflammatory nodules and abscess count with no increase in abscess and draining tunnels.^48^ In an open‐label cohort study of 10 moderate‐to‐severe HS patients, 100% of patients achieved HiSCR at Week 12, with significant improvements observed in pain, itch, and QoL endpoints.^26^ A subsequent open‐label study by the same authors assessed the effect of weekly administration (compared with every 2 weeks) on hard‐to‐treat draining tunnels, and reported that weekly brodalumab resulted in the rapid reduction of draining tunnels at Week 4, although the clinical difference observed between dosing regimens at Week 24 was negligible.^25^ The results of a Phase II randomized trial of bimekizumab (NCT03248531), including 88 moderate‐to‐severe HS patients, were recently presented by *Jemec* et al., showing that bimekizumab demonstrated clinically meaningful improvements in HS disease.^28^ No new safety signals of blocking the IL‐17 pathway were identified in any of these reports.

This study met its primary endpoint, despite higher‐than‐expected variability and a higher placebo rate. Due to this higher‐than‐expected placebo effect, the relatively small sample size, and the lack of re‐randomization, the data from the extension period did not demonstrate trends in efficacy. CJM112 followed by placebo, however, showed a somewhat sustained response for several months post‐treatment until the end of the study, whereas the effect of CJM112 in patients originally treated with placebo was not evident in the extension part of the study. Our study results, therefore, would need to be validated in larger patient cohorts.

In summary, this study utilized ex vivo HS tissue biopsies to elucidate the role of the IL‐17A pathway in the pathogenesis of HS, and clinically validated the IL‐17A pathway in moderate‐to‐severe HS in a proof‐of‐concept Phase II clinical study using the anti‐IL‐17A antibody CJM112. Currently, four Phase III randomized, controlled clinical trials are ongoing, the results of which will affirm the role of anti‐IL‐17 therapy in moderate‐to‐severe HS (secukinumab: SUNSHINE [NCT03713619], SUNRISE, [NCT03713632]; bimekizumab: BE HEARD I [NCT04242446], BE HEARD II [NCT04242498]).

## AUTHOR CONTRIBUTIONS

Conceptualisation: GBEJ, FK, CL. Data curation: IR, PJ, TP, LR, GW, FK. Formal analysis, software, validation: IR, PJ, TP, LR, GW, FK. Investigation and methodology: ABK, EPP, FGB, JW, GBEJ. Writing – original draft: GBEJ, CL. Writing – review and editing: ABK, CL, EPP, FGB, JW, IR, PJ, TP, LR, GW, FK, GBEJ. Funding acquisition: CL.

## CONFLICT OF INTEREST

**IR**, **PJ**, **LR**, **GW**, **TP**, **FK**, and **CL** are the employees of Novartis. **ABK** is a consultant and investigator for Abbvie, Janssen, Eli Lilly, Novartis, Pfizer, and UCB; investigator for Incyte, Bristol Meyers Squibb and Anapyts Bio; consultant for Bayer, Ventyx, Moonlake, Concert, EvoImmune, receives fellowship funding from Janssen and Abbvie; and served as previous Board of Directors and Past President of the International Psoriasis Council, Board of Directors of the HS Foundation and Board of Directors for Almirall. **EPP** has served as an advisory board member, consultant, speaker, and/or received investigator‐initiated grants from AbbVie, Amgen, Astra Zeneca, Biogen, Boehringer‐Ingelheim, ChemoCentryx, Celgene, Eli Lilly, Fresenius, InflaRx, Kymera, Leo Pharma, Janssen‐Cilag, Novartis, Pfizer, Regeneron, UCB. **FGB** has received honoraria from Abbvie, Novartis, Janssen‐Cilag, UCB, and LEO Pharma for participation on advisory boards or as a speaker. His department received grants for participation as an investigator in a clinical study for Abbvie, Novartis, Janssen‐Cilag, UCB, Almirall, LEO Pharma, and InflaRx. **GBE** has received honoraria from Afyx, ChemoCentryx, Coloplast, Incyte, Inflarx, Kymera, LEO Pharma, Toosonix, Novartis, Boehringer Ingelheim, VielaBio, Sanofi, UCB, Janssen Cilag and Union therapeutics for participation on advisory boards and has received grants from Abbvie, CSL Behring, Boehringer‐Ingelheim, InflaRx, LEO Pharma, Novartis, UCB and Regeneron for participation as an investigator. He has also received unrestricted departmental grants from LEO Pharma and Novartis. **JW** has received honoraria from Abbvie, Boehringer‐Ingelheim, Bristol Meyers Squibb, Eli Lilly, Janssen, Pfizer, Regeneron/Sanofi, UCB, and Novartis for acting as a consultant or speaker and has also received grants from Amgen, Abbvie, Anaptybio, Boehringer‐Ingelheim, Bristol Meyers Squibb, ChemoCentryx, Dermira, Eli Lilly, Galderma, GSK, Janssen, Pfizer, Regeneron/Sanofi, UCB and Novartis.

## Supporting information


**Figure S1.** Transcriptional analysis. (A) Unbiased analysis of differentially expressed genes using IPA software identified the ‘Role of IL‐17A in Psoriasis’ as one of the top canonical pathways (*p*‐value 8.52E‐09). Highlighted genes are present in the DEG list, and the red colour reflects the fold‐change upregulation in disease. (B) IPA upstream regulator analysis of differentially expressed genes identifies several IL‐17 cytokines and receptors as activated regulators. DEG, differentially expressed genes; IL, interleukin; IPA, ingenuity pathway analysis.

**Figure S2.** Patient Disposition. CONSORT flow chart describing number of patients entering and completing treatment period 1 (TP1) and extension period 2 (EP2).

**Figure S3.** Mean Lesion Counts at Baseline and Over Time to Week 16 in CJM112 300 mg‐ and Placebo‐treated HS Patients. Line graphs demonstrating (A) total inflammatory lesions, (B) abscesses, (C) inflammatory nodules and (D) draining tunnels from Baseline to Week 16 in CJM112 300 mg and placebo‐treated HS patients. Error bars represent standard deviation. HS, hidradenitis suppurativa.

**Figure S4.** Graphical summary. Summary illustrating the key results of transcriptomics and immunohistochemical analysis, and proof of concept Phase 2 clinical trial. CD, cluster of differentiation; DLQI, Dermatology Life Quality Index; hsCRP, high sensitivity C‐reactive Protein; HS, hidradenitis suppurativa; IL, interleukin; PGA, Physician's Global Assessment; Th, T helper.

**Appendix S1.** Supplementary Methods.
**Table S1.** Patient Baseline Demographics and Disease Characteristics.
**Table S2.** Adverse Events by System Organ Class at Week 16.

**Video S1.** CJM112 in HS–Video Abstract–Exp Derm–Final.

## Data Availability

Novartis is committed to sharing with qualified external researchers, access to patient‐level data and supporting clinical documents from eligible studies. These requests are reviewed and approved by an independent review panel on the basis of scientific merit. All data provided is anonymized to respect the privacy of patients who have participated in the trial in line with applicable laws and regulations.
