# Supplementary figures and images for: IL‐17A is a pertinent therapeutic target for moderate‐to‐severe hidradenitis suppurativa: Combined results from a pre‐clinical and phase II proof‐of‐concept study
Source: Exp Dermatol. 2022 Aug 19;31(10):1522–32. doi: 10.1111/exd.14619 (PMC9804780; doi:10.1111/exd.14619)

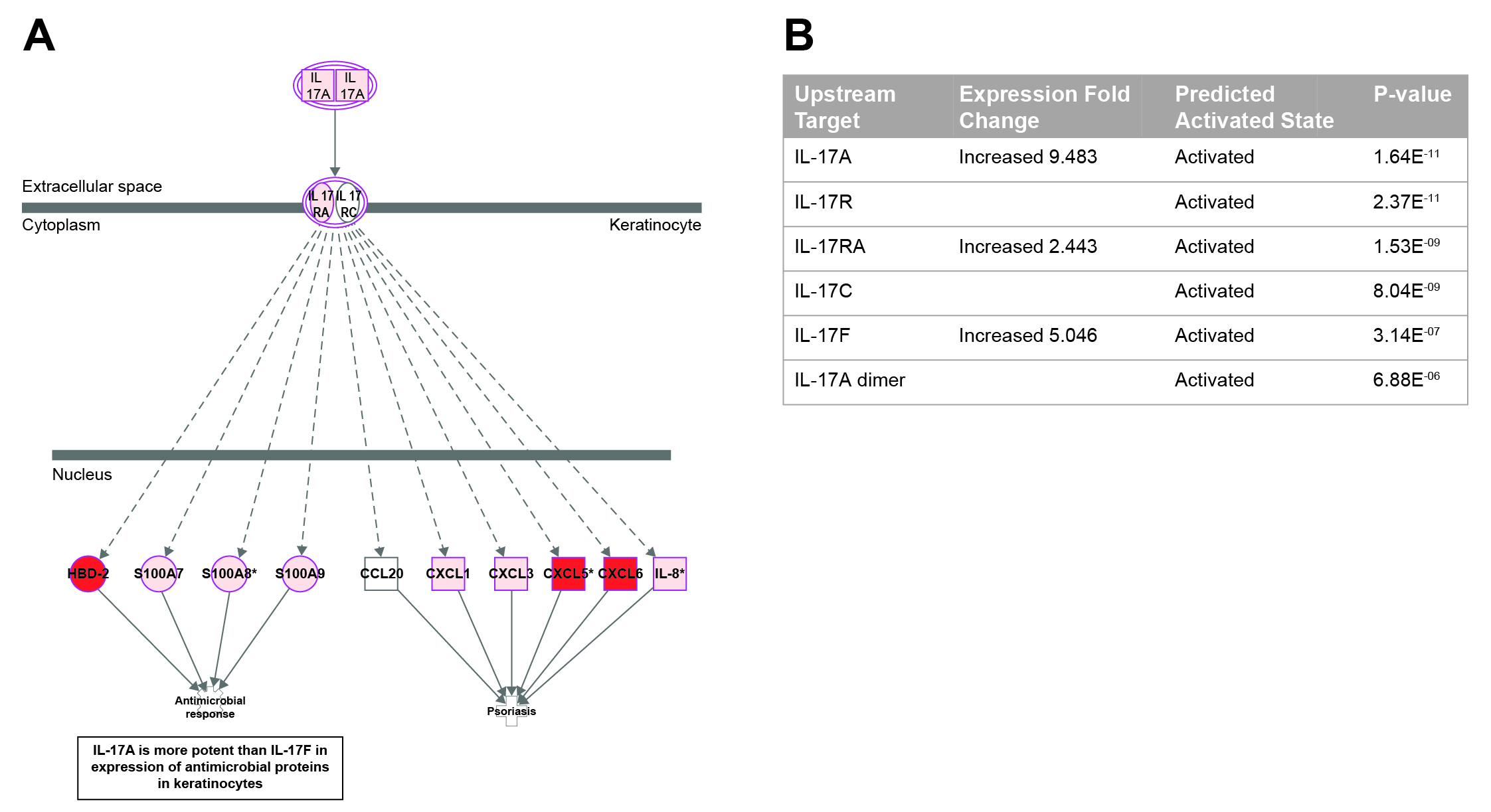

Supplement: Supplementary file 1 — Figure S1. Transcriptional analysis. (A) Unbiased analysis of differentially expressed genes using IPA software identified the ‘Role of IL‐17A in Psoriasis’ as one of the top canonical pathways (p‐value 8.52E‐09). Highlighted genes are present in the DEG list, and the red colour reflects the fold‐change upregulation in disease. (B) IPA upstream regulator analysis of differentially expressed genes identifies several IL‐17 cytokines and receptors as activated regulators. DEG, differentially expressed genes; IL, interleukin; IPA, ingenuity pathway analysis. [file EXD-31-1522-s006.jpg]

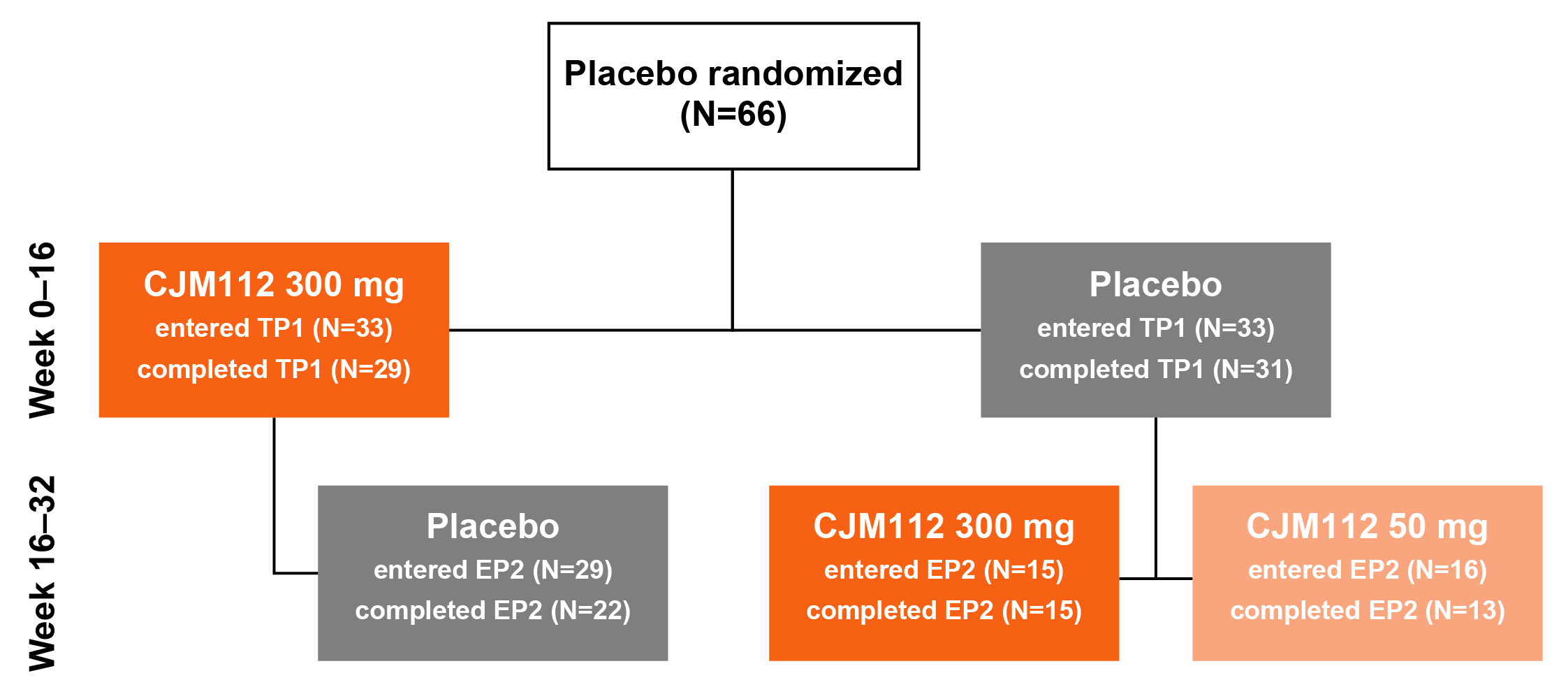

Supplement: Supplementary file 2 — Figure S2. Patient Disposition. CONSORT flow chart describing number of patients entering and completing treatment period 1 (TP1) and extension period 2 (EP2). [file EXD-31-1522-s001.jpg]

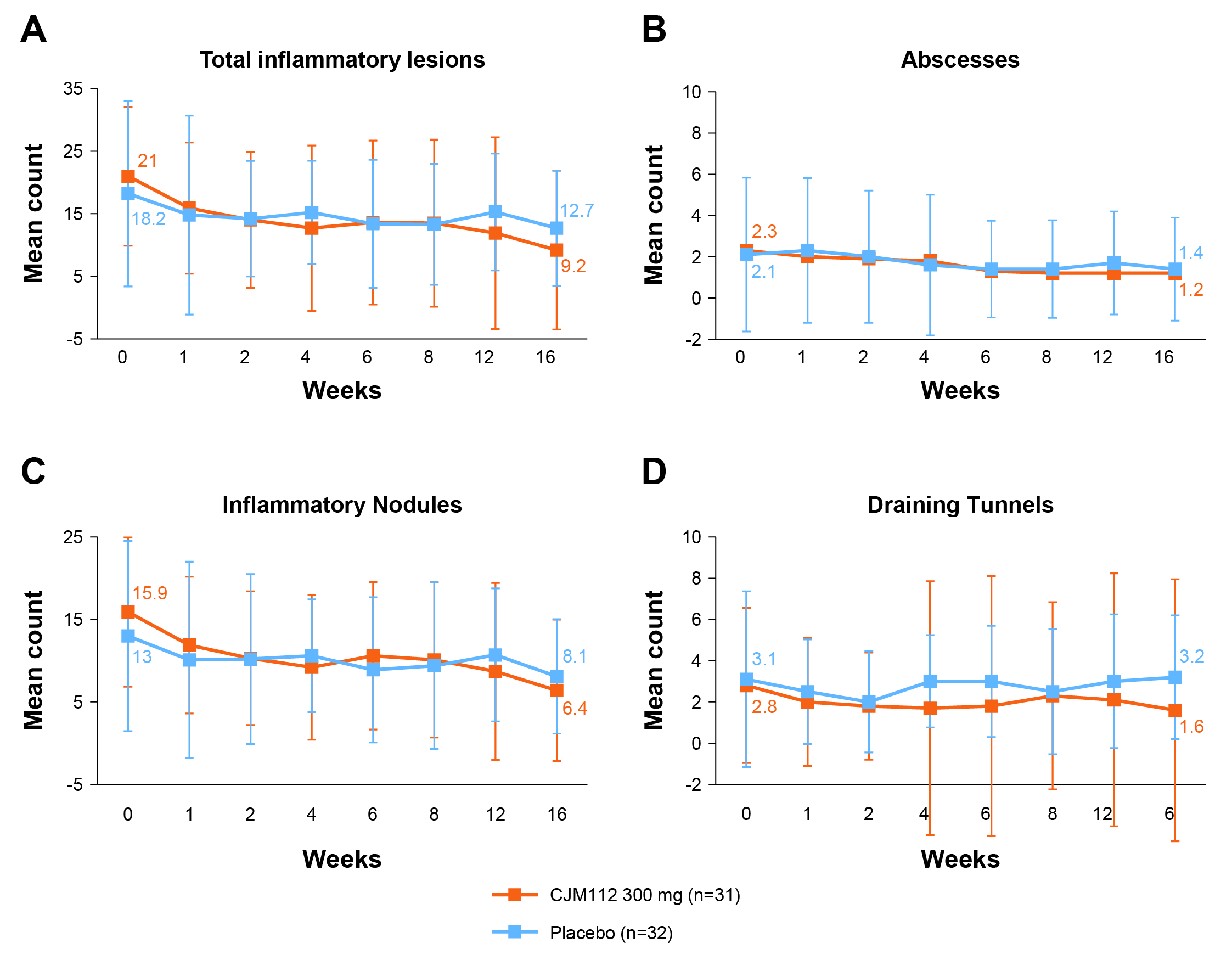

Supplement: Supplementary file 3 — Figure S3. Mean Lesion Counts at Baseline and Over Time to Week 16 in CJM112 300 mg‐ and Placebo‐treated HS Patients. Line graphs demonstrating (A) total inflammatory lesions, (B) abscesses, (C) inflammatory nodules and (D) draining tunnels from Baseline to Week 16 in CJM112 300 mg and placebo‐treated HS patients. Error bars represent standard deviation. HS, hidradenitis suppurativa. [file EXD-31-1522-s004.jpg]

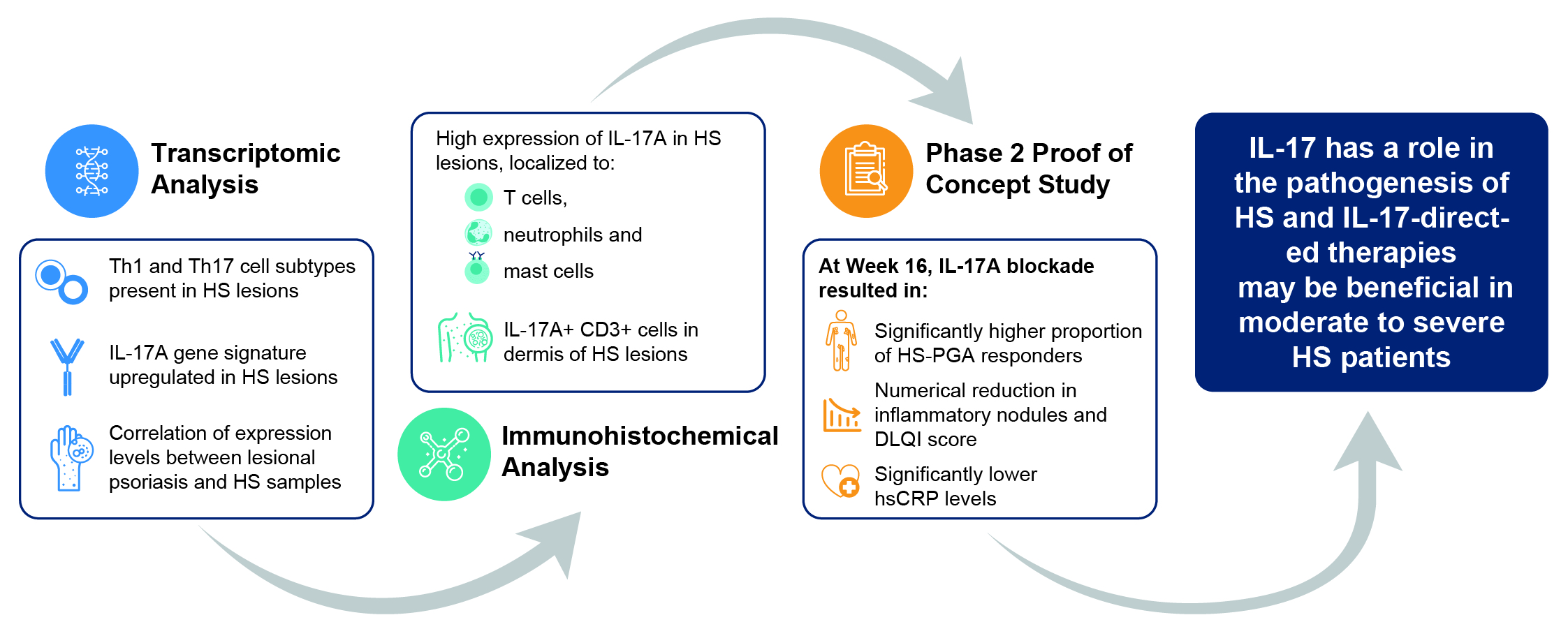

Supplement: Supplementary file 4 — Figure S4. Graphical summary. Summary illustrating the key results of transcriptomics and immunohistochemical analysis, and proof of concept Phase 2 clinical trial. CD, cluster of differentiation; DLQI, Dermatology Life Quality Index; hsCRP, high sensitivity C‐reactive Protein; HS, hidradenitis suppurativa; IL, interleukin; PGA, Physician's Global Assessment; Th, T helper. [file EXD-31-1522-s003.jpg]
