# Supplementary material for: IL‐17A is a pertinent therapeutic target for moderate‐to‐severe hidradenitis suppurativa: Combined results from a pre‐clinical and phase II proof‐of‐concept study
Source: Exp Dermatol. 2022 Aug 19;31(10):1522–32. doi: 10.1111/exd.14619 (PMC9804780; doi:10.1111/exd.14619)
Supplement: Supplementary file 5 — Appendix S1. Supplementary Methods. Table S1. Patient Baseline Demographics and Disease Characteristics. Table S2. Adverse Events by System Organ Class at Week 16. [file EXD-31-1522-s005.docx]

# TITLE: IL-17A is a pertinent therapeutic target for moderate to severe hidradenitis suppurativa: Combined results from a pre-clinical and Phase II proof-of-concept study

Running title: Anti-IL-17A therapy in moderate to severe HS

## **Authors:**

Alexa B Kimball, Christian Loesche, Errol P Prens, Falk G Bechara, Jamie Weisman, Izabela Rozenberg, Philip Jarvis, Thomas Peters, Lukas Roth, Grazyna Wieczorek, Frank Kolbinger, Gregor BE Jemec

# SUPPLEMENTARY INFORMATION

## List of supplementary information:

- Supplementary Methods
  - 1. Translational study
  - 2. Clinical study
- Supplementary Results
  - 2. Clinical study
- Supplementary Tables 1–2
- Supplementary Figure Legends 1–4

# SUPPLEMENTARY METHODS

# 1. TRANSLATIONAL STUDY

## 1.1 Collection of tissue samples for transcriptomics and immunohistochemical analysis

Surgical discard skin biopsies from chronic HS patients (n=19) and healthy volunteers (n=8) were obtained from the University Hospital Basel, Department of Plastic, Reconstructive & Aesthetic Surgery and Hand Surgery in Basel, Switzerland as part of a non-interventional biomarker study (TRI1270397). Visual assessment of patients at the time of surgery, as well as clinical and histopathological assessment, verified the diagnosis of patients included in the study. HS skin biopsies included affected anatomical areas with several disease states (inflammatory, and non-inflammatory, or fibrotic) from patients who underwent surgical removal of their affected skin, as well as skin biopsies of healthy control subjects who underwent cosmetic surgery. Psoriasis lesional skin was also used for transcriptomics analysis; these were obtained from commercial skin biopsy specimens from subjects with psoriasis (n=10; Asterand UK, Royston, United Kingdom). Biopsies used for transcriptomic profiling were stored in RNAlater (Thermo Fisher) at -80°C and biopsies used for immunohistochemical analysis were fixed in 10% Normal Buffered Formalin, and processed according to the standard procedure and embedded in paraffin.

## 1.2 Transcriptomic analysis by Affymetrix microarrays

From snap-frozen skin tissue sections, a homogenate was prepared using the recommended buffers from a Qiagen RNeasy mini kit (Qiagen, Hilden, Germany). Total RNA was extracted from the cells according to the manufacturer’s protocol. cDNA was then prepared from the same starting amount of RNA using a High Capacity cDNA Reverse Transcription Kit (Applied Biosystems, US). Samples were processed by CiToxLAB, Évreux, France on Affymetrix HG_U133_Plus2 microarrays. Robust Multichip Average (RMA) normalized data were analyzed using GeneSpring 11.5.1 (Agilent Technologies, Santa Clara, California, US) and the results were interpreted using Illumina BaseSpace Correlation Engine software and Qiagen Ingenuity Pathway Analysis (IPA).

Initially, the data were subject to standard quality control by CiToxLAB and in GeneSpring (Principal Component Analysis, hybridization controls). Subsequently, data were filtered on expression levels to probe sets above the 20^th^ percentile in 100% of the samples in any one of the conditions before further analysis. The normalized values were used to create a Spotfire file (TIBCO Spotfile Analyst 10.3.2) to calculate and visualize the average expression levels. To create differentially expressed gene lists, probe sets that were more that 2x and significantly different between the groups (i.e. lesional *vs*. healthy) were selected, using p<0.05 in an unpaired T Test and Benjamini-Hochberg False Discovery Rate (FDR).

Cell type deconvolution was performed using signatures from *Bindea et al.* (with the exception of Th17 cells, where a more extensive signature from *Ramesh et al.* was used) ^1,2^. These publications identified highly distinctive transcriptional profiles for the individual cell types based on Affymetrix data (approximately 20–50 genes per cell type). The cell type specific signatures were used to visualize the abundance of the cells in the HS dataset.

An IL-17A signaling signature was compiled based on IL‐17A stimulation and inhibition in different cell types, i.e., whole blood, keratinocytes, and/or synoviocytes as previously described ^3^. Members (genes) of this signature were consistently more highly expressed in lesional HS samples, indicating that IL-17A is active and induces signaling through its receptor in skin and infiltrating cell types. The HS microarray dataset from this study is available at National Center for Biotechnology Information Gene Expression Omnibus (NCBI GEO, accession number GSE148027).

In a focused RNA expression analysis (NanoString Inc, Seattle, Washington, US), the expression levels of a custom gene panel were compared between psoriasis (n=10) and HS lesional (n=8) samples. RNA isolated from psoriasis lesional skin samples were also analysed for comparison (as a known IL-17-driven disease which often co-occurs with HS^4-6^). For all psoriasis and HS samples, 50 ng of RNA was preamplified by using the Affymetrix SensationPlus^TM^ FFPE Amplification kit (Affymetrix, Santa Clara, Calif). Preamplified RNA samples were processed with the nCounter Prep Station and Digital Analyzer of NanoString Technologies. The custom-designed nCounter Gene Expression CodeSets C1933_Amadeus and C2018_Everest/C2489_ Chomolungma overall contained probe sets for 314 target transcripts, 7 candidate reference genes for normalization, and 2 gender control genes. Three hundred nanograms (C1933_Amadeus) or 25 ng (C2018_Everest or C2489_ Chomolungma) of the preamplified sense RNA was hybridized with the respective CodeSet at 65°C for 16 hours. Post-hybridization processing procedures were carried out, as recommended by NanoString Technologies. Cartridges were scanned at a resolution of 600 fields of view. Gene expression barcode counts were analyzed with nSolver Analysis software v1.1 (NanoString Technologies). Raw barcode counts were normalized lane-to-lane using the geometric mean of counts obtained for six spike-in exogenous positive control probe sets in the CodeSet, and then by using the geometric mean of the technically normalized counts of three endogenous genes (*RPL13A*, *RPL19*, and *UBC*). The psoriasis-based gene set showed a good correlation of expression levels between the two diseases. Specific genes of interest identified as upregulated in psoriasis and HS tissue were then further validated using Affymetrix data.

## 1.3 Immunohistochemical analysis of HS skin biopsies

Fourteen skin biopsies (seven lesional and seven non-lesional) from five HS patients and two skin biopsies from healthy volunteers, were included in the immunohistochemical analysis. Paraffin sections of skin biopsies (3 μm thick) from HS patients and healthy volunteers were cut and stained with hematoxylin and eosin. Automated immunohistochemical stainings for myeloperoxidase (neutrophils) (MPO; rabbit polyclonal ab45977, Abcam, UK), CD68 (macrophages) (clone KP-1, Dako, Denmark), CD3 (T cells) (Clone: SP7, Thermo Fisher Scientific, UK), CD20 (B cells) (Clone: L26, Dako, Denmark), β-defensin 2 (BD-2) (goat polyclonal 500-P161G, Peprotech, USA), S100A7/Psoriasin (Clone: 47C1068, Novus Biologicals, UK) and IL-17A (goat polyclonal AF-317-NA, R&D Systems, UK) were performed on Ventana Discovery XT immunostainer (Roche Diagnostics, Switzerland). Specific isotype controls were used as negative controls. All biopsy samples were digitalized using ScanScope XT slide scanner (Aperio, Leica Biosystems, Switzerland) at x40 magnification.

Dual immunofluorescence staining was performed manually after 20 minutes of heat-induced epitope retrieval in citrate buffer, pH6 at 98˚C. Incubation of primary antibodies, anti-CD3 and anti-IL-17A, were followed by secondary antibody chicken anti-rabbit Alexa488 and chicken anti-goat Alexa 594 (Invitrogen, Thermo Fisher Scientific, UK). Sections were mounted with ProLong Antifade Gold with 4,6-diamidino-2-phenylindole (DAPI; Invitrogen). The confocal images were acquired using LSM 700 confocal microscope (Zeiss, Germany).

# 2. CLINICAL STUDY

## 2.1 Patients and inclusion/exclusion criteria

Adult patients aged between 18–65 years and weighing between 50–150 kg with chronic moderate to severe HS for at least 1 year (prior to screening), who had previously undergone antibiotic therapy were included in this study. Moderate to severe HS was defined as having a HS-PGA score of at least moderate severity (score ≥3) and with at least 4 abscesses and/or nodules. Additional inclusion criteria required patients to have HS lesions in at least two anatomical areas and at least one area had to be minimally Hurley stage II (moderate). Patients receiving systemic treatment (including retinoids or other immunomodulating therapies) or antibiotics were required to stop treatment at least 4 weeks before randomization/first treatment. Patients previously treated with IL-17 or IL-17R blockers including secukinumab, ixekizumab and brodalumab were excluded from the study. Adequate wash out periods were applied for patients treated with biologics; no wash out period was requested for treatment with topicals. A further list of inclusion and exclusion criteria can be found at [clinicaltrials.gov, NCT02421172](https://clinicaltrials.gov/ct2/show/NCT02421172).

**2.2 Sample size calculation**

A final group size of 30 patients in both the CJM112 300 mg and placebo groups assured the minimal probability to reach the target efficacy, based on the assumptions that the placebo HS-PGA responder rate was around 5% (based on Kimball et al. 2012^7^) and the CJM112-placebo difference of the HS-PGA responder rates was at least 30%. As a pilot study, sample size calculations were not carried out for secondary or exploratory efficacy endpoints.

## 2.3 Statistical analysis

The HS-PGA responder rate is a binary outcome, therefore Bayesian inference based on non-informative prior Beta (1/3, 1/3) for each group was used to compare HS-PGA responder rates between CJM112 300 mg and placebo groups at Week 16. Target efficacy was achieved if the posterior distribution for the difference in CJM112 300 mg treatment HS-PGA responder rate (placebo HS-PGA responder rate) had a 90% probability of being positive and if a moderate confidence (60% probability) of the difference in HS-PGA responder rates was greater than 30%. Estimates of the posterior probabilities of the difference of the HS-PGA responder rate between the CJM112 30 mg and placebo groups at Week 16 are presented together with 95% confidence intervals.

# SUPPLEMENTARY RESULTS

# 2. CLINICAL STUDY

## 2.1 Pharmacokinetics and Pharmacodynamics of CJM112

The mean elimination of CJM112 was as expected for an IgG1 antibody with a mean elimination half-life of 16–20 days, and no dose dependency. Target engagement was demonstrated through a saturable accumulation of drug-target complexes (accumulation of the CJM112-IL-17A complexes) in serum. Despite some inter-individual variability in the total IL-17A profiles, these profiles were characteristic of a slow elimination of the CJM112-IL-17A complex compared to free IL-17A (taking on a slow elimination of CJM112), a slow turnover of IL-17A and a long duration of IL-17A capture.

## 2.2 Safety

In total, 55/66 patients (83.3%) reported at least one AE at Week 16, with overall incidence similar between CJM112 300 mg and placebo groups (**Table 2**). The most common AEs by system organ class (SOC) were infections and infestations (54.5%), gastrointestinal disorders (33.3%), skin and subcutaneous tissue disorders (27.3%), general disorders and administration site conditions (24.2%), musculoskeletal and connective tissue disorders (22.7%). The most common AEs were nasopharyngitis (16.7%), nausea (12.1%), diarrhea (10.6%) and headache (10.6%). Among these, nasopharyngitis and nausea incidences were higher in the CJM112 300 mg group compared to placebo. Of note, two patients in both the CJM112 300 mg group and placebo group experienced a fungal infection (CJM112 300 mg; cryptosporidiosis infection and skin candida, placebo: fungal infection and vulvovaginal mycotic infection). Similar patterns of AEs were observed from Week 16–32 (**data on file**). Overall, five patients were discontinued from this study due to an AE (1 patient between Weeks 0–16, 4 patients between Weeks 16–32).

One patient receiving CJM112 300 mg in TP1 experienced cystitis which was considered not to be study drug related. The second patient (CJM112 300 mg/placebo in EP2) reported non-serious hypersensitivity vasculitis (two episodes on Day 150 and Day 169). This patient had pre-existing purpura on the lower legs which worsened after the Week 16 visit. A skin biopsy was taken which led to the diagnosis of hypersensitivity. Three discontinuations due to AEs were considered related to study drug (all CJM112 300 mg/placebo in EP2). One patient reported pneumonia 8 days after receiving the last CJM112 300 mg dose. Another patient reported blurred vision, dizziness and headaches 14 days after receiving the last dose of CJM112 300 mg. A third patient was discontinued on Day 157 due to shivering which occurred a day after receiving the last CJM112 300 mg dose.

# SUPPLEMENTARY TABLES

|  | **CJM112 300 mg**  **N=33** | **Placebo**  **N=33** | **Total**  **N=66** |
| --- | --- | --- | --- |
| **Age (years),** mean±SD | 36±9.8 | 39±10.9 | 37±10.5 |
| **Sex, female,** n (%) | 22 (66.7) | 22 (66.7) | 44 (66.7) |
| **Race,** n (%)  Caucasian  Black  Other | 22 (66.7)  8 (24.2)  3 (9.1) | 21 (63.6)  8 (24.2)  4 (12.1) | 43 (65.2)  16 (24.2)  7 (10.6) |
| **Weight (kg),** mean±SD | 94.7±22.19 | 94.8±21.59 | 94.8±21.72 |
| **BMI (kg/m^2^),** mean±SD | 32.0±7.19 | 33.3±7.49 | 32.7±7.32 |
| **Hurley score,** n (%)  Stage II  Stage III | 14 (42)  19 (58) | 20 (61)  13 (39) | 34 (52)  32 (48) |
| **HS-PGA score,** mean±SD | 3.9±0.86 | 3.8±0.91 | 3.9±0.88 |
| **HS-PGA score,** n (%)  Moderate  Severe  Very severe | 14 (42.4)  9 (27.3)  10 (30.3) | 16 (48.5)  6 (18.2)  11 (33.3) | 30 (45.5)  15 (22.7)  21 (31.8) |
| **Overall inflammatory lesion count,** mean±SD | 20.6±14.55 | 18.0±10.99 | 19.3±12.86 |
| Abscesses | 2.2±3.64 | 2.0± 2.74 | 2.1± 3.20 |
| Inflammatory nodules | 15.5±11.31 | 12.9±8.92 | 14.2±10.19 |
| Draining tunnels | 2.9±4.22 | 3.0±3.72 | 3.0±3.95 |
| **hsCRP (mg/L),** mean±SD | 19.2±22.55 | 21.0±17.51 | 20.1±20.05 |
| **DLQI score,** mean±SD | 19.8±7.3 | 16.5±8.0 | 18.1±7.8 |

**Supplementary Table 1. Patient Baseline Demographics and Disease Characteristics.** BMI, Body Mass Index; DLQI, Dermatology Life Quality Index; hsCRP, high sensitivity C-Reactive Protein; HS-PGA, Hidradenitis Suppurativa - Physician Global Assessment; Kg, kilogram; SD, standard deviation.

**Supplementary Table 2. Adverse Events by System Organ Class at Week 16.**

|  | **CJM112 300 mg**  **N=33** | **Placebo**  **N=33** | **Total**  **N=66** |
| --- | --- | --- | --- |
| **Patients with AE** | 28 (84.8) | 27 (81.8) | 55 (83.3) |
| **System Organ Class** |  |  |  |
| Infections and infestations | 18 (54.5) | 18 (54.5) | 36 (54.5) |
| Gastrointestinal disorders | 12 (36.4) | 10 (30.3) | 22 (33.3) |
| Skin and subcutaneous tissue disorders | 9 (27.3) | 9 (27.3) | 18 (27.3) |
| General disorders and administration site conditions | 8 (24.2) | 8 (24.2) | 16 (24.2) |
| Musculoskeletal and connective tissue disorders | 9 (27.3) | 6 (18.2) | 15 (22.7) |
| Respiratory, thoracic and mediastinal disorders | 5 (15.2) | 5 (15.2) | 10 (15.2) |
| Nervous system disorders | 6 (18.2) | 3 (9.1) | 9 (13.6) |
| Investigations | 3 (9.1) | 5 (15.2) | 8 (12.1) |
| Injury, poisoning and procedural complications | 3 (9.1) | 3 (9.1) | 6 (9.1) |
| **Preferred Term*** |  |  |  |
| Nasopharyngitis | 7 (21.2) | 4 (12.1) | 11 (16.7) |
| Nausea | 5 (15.2) | 3 (9.1) | 8 (12.1) |
| Diarrhea | 2 (6.1) | 5 (15.2) | 7 (10.6) |
| Headache | 4 (12.1) | 3 (9.1) | 7 (10.6) |
| Back pain | 5 (15.2) | 0 | 5 (7.6) |
| Oropharyngeal pain | 2 (6.1) | 3 (9.1) | 5 (7.6) |
| Pruritus | 3 (9.1) | 2 (6.1) | 5 (7.6) |
| Fatigue | 3 (9.1) | 1 (3.0) | 4 (6.1) |
| Influenza | 3 (9.1) | 1 (3.0) | 4 (6.1) |
| Pyrexia | 3 (9.1) | 1 (3.0) | 4 (6.1) |
| Toothache | 1 (3.0) | 3 (9.1) | 4 (6.1) |
| Urinary tract infection | 2 (6.1) | 2 (6.1) | 4 (6.1) |

*Frequency of >5% in total group. AE, adverse event

# SUPPLEMENTARY FIGURE LEGENDS

**Supplementary Figure 1: Transcriptional analysis. (A)** Unbiased analysis of differentially expressed genes using IPA software identified the “Role of IL-17A in Psoriasis” as one of the top canonical pathways (p-value 8.52E-09). Highlighted genes are present in the DEG list and the red color reflects the fold-change upregulation in disease. **(B)** IPA upstream regulator analysis of differentially expressed genes identifies several IL-17 cytokines and receptors as activated regulators. DEG, differentially expressed genes; IL, interleukin; IPA, Ingenuity Pathway Analysis.

**Supplementary Figure 2: Patient Disposition.** CONSORT flow chart describing number of patients entering and completing treatment period 1 (TP1) and extension period 2 (EP2).

**Supplementary Figure 3: Mean Lesion Counts at Baseline and Over Time to Week 16 in CJM112 300 mg- and Placebo-treated HS Patients.** Line graphs demonstrating **(A)** total inflammatory lesions, **(B)** abscesses, **(C)** inflammatory nodules and **(D)** draining tunnels from Baseline to Week 16 in CJM112 300 mg and placebo treated HS patients. Error bars represent standard deviation. HS, hidradenitis suppurativa.

**Supplementary Figure 4: Graphical summary.** Summary illustrating the key results of transcriptomics and immunohistochemical analysis, and proof of concept Phase 2 clinical trial. CD, cluster of differentiation; DLQI, Dermatology Life Quality Index; hsCRP, high sensitivity C-reactive Protein; HS, hidradenitis suppurativa; IL, interleukin; PGA, Physician’s Global Assessment; Th, T helper.

# References

1. Ramesh R, Kozhaya L, McKevitt K, et al. Pro-inflammatory human Th17 cells selectively express P-glycoprotein and are refractory to glucocorticoids. *J Exp Med.* 2014;211(1):89-104.

2. Bindea G, Mlecnik B, Tosolini M, et al. Spatiotemporal dynamics of intratumoral immune cells reveal the immune landscape in human cancer. *Immunity.* 2013;39(4):782-795.

3. van Tok MN, van Duivenvoorde LM, Kramer I, et al. Interleukin-17A Inhibition Diminishes Inflammation and New Bone Formation in Experimental Spondyloarthritis. *Arthritis & rheumatology (Hoboken, NJ).* 2019;71(4):612-625.

4. Kjaersgaard Andersen R, Saunte SK, Jemec GBE, Saunte DM. Psoriasis as a comorbidity of hidradenitis suppurativa. *Int J Dermatol.* 2020;59(2):216-220.

5. Kridin K, Shani M, Schonmann Y, et al. Psoriasis and Hidradenitis Suppurativa: A Large-scale Population-based Study. *J Am Acad Dermatol.* 2018.

6. Pinter A, Sarlak M, Zeiner KN, et al. Coprevalence of Hidradenitis Suppurativa and Psoriasis: Detailed Demographic, Disease Severity and Comorbidity Pattern. *Dermatology.* 2020:1-10.

7. Kimball AB, Kerdel F, Adams D, et al. Adalimumab for the treatment of moderate to severe Hidradenitis suppurativa: a parallel randomized trial. *Ann Intern Med.* 2012;157(12):846-855.
